# Investigating factors influencing recruitment and retention of adult community nurses: an exploratory qualitative study across NHS trusts in England

**DOI:** 10.1017/S1463423625100480

**Published:** 2025-10-08

**Authors:** Edwin Chamanga, Judith Dyson, Manuela Jarrett, Eamonn McKeown

**Affiliations:** 1 City, University of London, London, UK; 2 Birmingham City University, Birmingham, UK; 3 University of Birmingham, Birmingham, UK

**Keywords:** Attrition, Community nursing, Herzberg two actor theory, Recruitment, Retention

## Abstract

**Aim:**

To investigate factors influencing the recruitment and retention of adult community nurses.

**Background:**

The recruitment and retention of community nurses is a growing global challenge, exacerbated by aging populations and increasing demand for primary and home-based care. Across Europe, nurse shortages threaten healthcare sustainability, with high attrition rates linked to workplace pressures, inadequate staffing, and emotional exhaustion. Despite efforts to strengthen retention, many European countries struggle to maintain adequate staffing levels, particularly in community nursing.

**Methods:**

An exploratory qualitative approach was used with semi-structured interviews. The interview schedule was shaped by the study’s aims, a prior integrative literature review, and the ‘causal model of turnover for nurses’. Questions explored participants’ experiences of recruitment into community nursing and factors influencing retention. The study focused on registered nurses and service managers within adult community nursing organizations across diverse geographical areas.

**Findings:**

The study identified eight main themes influencing recruitment and retention: the perfect job, finding true self and fulfilment, alignment with organizational values, prior development and transitional experience, job dissatisfaction, shift in traditional practices, lack of compassionate leadership, and family commitments. Key factors included workplace flexibility, professional identity, job security, and organizational culture. However, challenges such as staffing shortages, lack of career progression, and increased administrative tasks were significant barriers to retention.

**Conclusion:**

This study highlights the multifaceted challenges surrounding community nurse recruitment and retention, emphasizing the need for targeted strategies that go beyond traditional hospital-focused approaches. While salary improvements remain crucial, broader systemic changes including workplace flexibility, compassionate leadership, and career development opportunities are essential to fostering a sustainable workforce. By addressing these factors through co-designed solutions and evidence-based policy adjustments, healthcare organizations can enhance job satisfaction, reduce attrition, and ultimately strengthen the future of community nursing.

## Introduction

The recruitment and retention of community nurses is a growing global challenge, exacerbated by aging populations and increasing demand for primary and home-based care (World Health Organization (WHO, 2008; Hajat and Kishore, 2018). Across Europe, nurse shortages threaten healthcare sustainability, with high attrition rates linked to workplace pressures, inadequate staffing, and emotional exhaustion (Estryn-Behar et al., 2010; Maurits et al., 2015). The European Federation of Nursing Associations warns that policy gaps and funding limitations have left healthcare systems vulnerable, urging investment in workforce resilience (De Raeve, 2022).

Despite efforts to strengthen retention, many European countries struggle to maintain adequate staffing levels, particularly in community nursing (WHO, 2024). Research highlights that workplace conditions, career progression, and leadership support are critical to nurse retention, yet remain underdeveloped in many healthcare systems (Chamanga et al., 2020).

### The UK perspective

In England, community nursing teams provide essential care for older adults, yet workforce shortages persist (Queen’s Nursing Institute [QNI], 2014). While nurse recruitment strategies are being implemented, retention remains a pressing issue, with policies overwhelmingly focused on acute care rather than community settings (Royal College of Nursing [RCN], 2013; Maybin et al., 2016). Evidence suggests that retention strategies often fail due to a lack of understanding of why nurses choose to stay, leading to increased reliance on costly agency staff (Health Education England [HEE, 2014]; The Health Foundation and The Nuffield Trust, 2018).

Community nurses experience higher work pressure than their hospital-based counterparts, with overstretch and burnout contributing to attrition (Marangozov et al., 2017). Reports highlight a workforce crisis, as demand outpaces capacity, further straining healthcare delivery (Maybin et al., 2016).

### Study significance

Despite record recruitment levels, community nursing numbers continue to decline, likely due to hospital-centric workforce models (Marufu et al., 2021; Morris et al., 2023). Existing retention strategies often focus on why nurses leave, rather than why they stay, reducing effectiveness (Loan-Clarke et al., 2010; Health Education England [HEE, 2014]). The National Health Service (NHS) reliance on agency nurses rose by 10% in 2017/18, costing over £5.5 billion, diverting resources from care improvement (The Health Foundation, The King’s Fund & The Nuffield Trust, 2018).

This study seeks to fill the gap by qualitatively exploring community nurse recruitment and retention, examining professional identity, workplace flexibility, organizational values, and leadership (Chamanga et al., 2020; Marufu et al., 2021). Addressing these concerns through evidence-based strategies is crucial for sustaining the workforce and informing policy adjustments.

### The study

#### Aim

To investigate factors influencing the recruitment and retention of adult community nurses.

## Methods

### Study design

This study employed an exploratory qualitative design (Ritchie et al., 2013) using semi-structured interviews to examine community nursing recruitment and retention. Given the limited existing knowledge, this approach allowed open-ended exploration, free from preconceived explanations. The design was fluid, flexible, data-driven, and systematic, enabling rich data collection in natural settings, starting from individuals’ expressions and activities while embracing diversity.

The ‘causal model of turnover for nurses’ (Price and Mueller, 1981) informed the interview process, identifying key determinants based on longitudinal data from 1,091 registered nurses across various healthcare settings. This guided the structured yet adaptive questioning framework. Data analysis followed the framework approach (Gale et al., 2013), ensuring a systematic yet flexible qualitative research process, enabling researchers to categorize findings into an analytical framework using NVivo 12®.

### Study setting

The study was conducted within NHS Trusts across England, strategically selected to capture diverse recruitment and retention experiences across urban, rural, and coastal settings (de Chesnay, 2016). Selection criteria included Care Quality Commission (CQC) ratings, geographical diversity, and recruitment trends based on vacancy percentages.

### Study participants and recruitment

A purposive sampling strategy was employed, selecting participants across various NHS Trust roles to ensure relevant experience with recruitment and retention of adult community nurses. This two-level sampling approach maximized diversity and richness in perspectives. Demographic data supported analysis but were not used for initial sampling. Purposive sampling ensured relevant representation, avoiding convenience sampling bias.

### Inclusion and exclusion criteria

Inclusion criteria covered qualified nurses registered with the Nursing and Midwifery Council (NMC) working in adult community NHS Trusts in England, alongside service managers, regardless of contract type. Exclusion criteria omitted nurses in general practice, hospital outreach roles, Nursing Associates, and Healthcare Assistants. Recruitment was facilitated via Research and Development (R&D) leads, with invitations sent via email. Consent was obtained through scanned wet-signature forms, and reminders targeted underrepresented staff groups.

### Interview process

Interviews were conducted by EC, a male clinical nurse with research training, known to few participants. Sessions were held via Microsoft Teams, allowing convenience for geographically dispersed individuals from July 2020 to February 2021. Discussions were recorded and transcribed verbatim, with durations ranging from 32 to 75 minutes, averaging 41 minutes.

### Ethical considerations and reflexivity

**Ethical Statement:** Participant consent was obtained via a consent form with a wet signature.

Ethical approval was granted by City, University of London School of Health Sciences Research Ethics Committee and NHS Health Research Authority (IRAS project ID: 264073). Informed consent procedures ensured participant autonomy, allowing withdrawal without explanation. Confidentiality was upheld except in cases of harm or intent to harm.

Reflexivity was integral, acknowledging researchers’ backgrounds in community nursing and management, recognizing potential biases while leveraging professional insights. To enhance qualitative rigour, the study followed the Consolidated Criteria for Reporting Qualitative Studies (COREQ) 32-item checklist (Tong et al., 2007), ensuring transparency, reliability, and depth in reporting.

## Findings

### Characteristics of participants

Table 1 presents the characteristics of the diverse organizations included in the study, reflecting variations in organizational and population demographics.

**Table 1.** Characteristics of participant community nursing organizations

Characteristics		Urban trust	Rural trust	Coastal trust
Type of organization		Standalone	Vertically integrated^a^	Standalone
Population size		300,000	450,000	1,500,000
Local population ethnic makeup %	Black African/ Caribbean	6.5	0.3	1.1
	Asian	34.4	2.7	3.3
	White	51.4	96.0	93.7
	Mixed heritage	4.1	0.7	1.5
	Other	3.5	0.2	0.5
Staff size		1,200	6,800	5,700
Vacancy rate %		22	21	21.3
Independent regulatory body rating		Good	Requires improvement	Outstanding
Geographical coverage		An urban area of a major city	Multiple villages and six towns	Multiple villages and a city
Life expectancy in years	Men	80	79	79
	Women	83	82.4	83.2

^a^The adult community nursing organization is part of an acute Trust.

Table 2 summarizes participants’ characteristics, grouped broadly to maintain anonymity. Participants are assigned pseudonyms along with their roles. The study included 50 individuals: 13 in senior management or non-clinical roles (e.g. service managers, clinical practice facilitators) and 37 clinical community nurses. Participants varied in seniority levels and were predominantly women (n = 45), with four on temporary contracts. Pseudonyms were assigned to all participants to ensure anonymity.

**Table 2.** Summary of participant characteristics according to geographical location

Characteristics		Urban	Rural	Coastal
Nature of role	Clinical	16	13	8
	Manager	5	4	4
Age of staff	20–30	1	0	1
	31–40	2	7	5
	41–50	8	6	2
	51–60	10	4	3
	61–70	0	0	1
Ethnicity	Black African	11	0	0
	Caribbean	1	0	0
	Chinese	1	0	0
	Indian	1	0	0
	Filipino	1	0	1
	Polish	1	0	0
	White British	4	17	10
	White Irish	1	0	1
Years in community practice	0–5	7	1	5
	6–10	4	8	2
	11–15	4	2	0
	16–20	3	4	3
	21–25	2	0	1
	26–30	1	0	0
	>31	0	2	1
Gender	Male	3	1	0
	Female	17	16	12
	Declined	1	0	0
Contract	Permanent	18	17	11
	Temporary	3	0	1
Role	District nurse	7	4	4
	Community staff nurse	5	2	3
	Service manager	3	3	4
	Community matron	2	3	0
	Specialist Nurse	2	4	1
	Clinical practice facilitator	2	1	0

**Figure 1.**
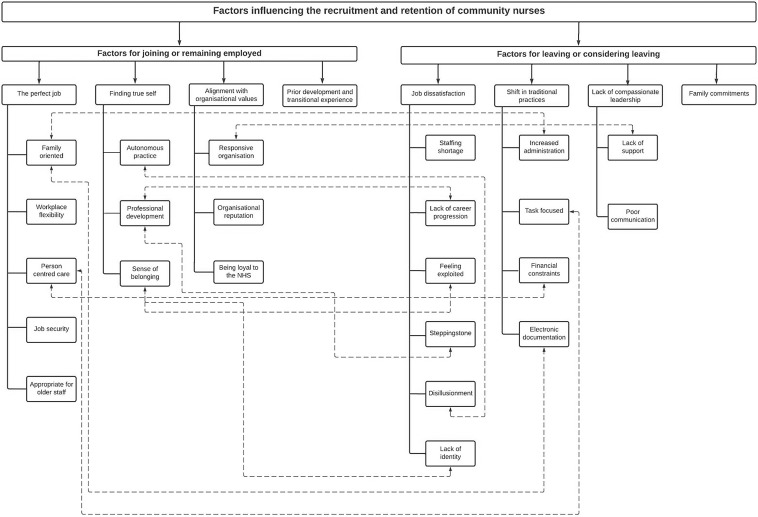
Factors that influence the recruitment and retention of community nurses.

Eight main themes with 23 sub-themes are illustrated in Figure 1 and presented sequentially. Themes are structured around recruitment (motivation factors) and retention (hygiene factors), when applying Herzberg two factor theory. As some findings highlight factors that contribute to recruitment, while their absence leads to attrition but not necessarily sequential but over a period. Differences in experiences between participant groups (e.g. by role) are incorporated into the narrative.

### Theme 1: the perfect job

Participants viewed community nursing as the perfect job, providing purpose and control over work and personal life. They seamlessly integrated work into their home lives, emphasizing the importance of family life and comfort in being away from work without feelings of guilt.

#### Family oriented

Participants, across all roles and contract types, viewed community nursing as family oriented, allowing nurses to manage family demands, sometimes during work hours as well as after work. This was facilitated by working close to home and during the day without night shifts.

*‘Getting married and having kids, because it was easier for me to have a job in the community…’* (Ellis, community matron).

#### Workplace flexibility

Participants with permanent contracts and service managers, believed workplace flexibility and informal local arrangements were a reason for community nurses to join or remain employed in community nursing. All three community nursing organizations reported having policies supporting workplace flexibility, characterized by changes in contracted working hours or working patterns to accommodate personal circumstances.

*‘… If I am flexible it will make me stay where I am because of the flexibility I get’* (Jay, community staff nurse)

*‘So at interview, we always discuss the hours of service and what service we offer … just because we advertise it as a full time doesn’t mean that we will only consider full time’* (Lou, service manager).

#### Person centred care

Many participants valued the interpersonal relationships in community nursing, extending from individualized care planning to patients as part of a wider community. They enjoyed co-designing care plans with patients in their homes, where they were guests but respected as experts.

*‘ … the rapport that I established with them…making a plan with them…show it to them and ask them to read it’.* (Ruth, district nurse)

#### Job security

Some participants said job security in a state-funded healthcare organization influenced their decision to apply or remain in the job. They viewed their organizations as reliable offering financial security. This was not only the case for permanent nurses, temporary contract community nurses, linked their job security to their in-demand skills and knowledge in community nursing.

*‘…people coming into the health economy, they do get some stability and some security in the role, so we have been so lucky you know that we have been continued to be employed and to be paid …’* (Maggie, service manager).

#### Appropriate for older staff

Participants, in particular those aged over 41, from all sites viewed community nursing as a suitable career for older individuals. The concept of ‘older’ varied, ranging from nearing retirement to self-perceived ageing. This was often linked to the inability to meet the physical demands of hospital work, prompting consideration of community nursing.

‘*I started to find that the confines of the hospital system, you know, I wanted a more relaxed approach as I got older’.* (Ella, specialist nurse)

An older workforce was not always considered positive in relation to attrition due to retirement, however some areas had strategies in place to address this.

*‘…we have a workforce that’s old … an average a year, we get about four retirements, which is quite significant impact on the on the service. And the one way, one thing that we’ve done to combat that is to offer retire and return contracts…’* (Alex, service manager).

### Theme 2: finding true self, fulfilment

This theme included references to participants feelings of personal satisfaction, growth and contentment within their community nursing roles.

#### Autonomous practice

Most participants, regardless of their contracts and roles, identified autonomous practice as a unique aspect of community nursing that attracted them to the field. They talked about their freedom and ability to make independent clinical decisions based on their scope of practice and experience. Nurses enjoyed the freedom to identify multiple conditions, prescribe treatments, and promote self-management and self-care right in the patients’ homes.

*‘Being able to actually make a difference to patients … prevent hospital admissions … having those skills to manage complex patients at home’* (Gerry, district nurse).

There was a sentiment that a reduction in autonomy could lead to community nurses leaving their roles. Early hospital discharge was seen as a driver for community nurses to develop advanced nursing skills. Service managers believed this enhanced autonomy of practice.

#### Professional development

Participants appreciated the development opportunities available to community nurses. This included access to continuous professional development and career advancement opportunities. It also included secondments and becoming link champions for specialist nursing roles.

*‘…I thought you know what I’ll do that, and I did the physical assessment, and I did the clinical decision making and then I was the first in the Trust to complete the prescribing course’.* (Drew, community matron)

#### Sense of belonging

Most participants with permanent contracts and one with a temporary contract reported feeling a sense of belonging within their teams and the broader organization. This sense of belonging was defined in various ways, such as viewing the role as a vocation, considering the team an extended family or seeing it as an informal support network.

*‘The reason I’ll stick with this community is because I work with an extremely supportive, fantastic team of people. They know more about me than my family do. We are very close, and we look after each other…’* (Nicky, specialist nurse).

Managers particularly spoke about the value of social activities, such as Christmas parties and birthday celebrations, in enhancing the sense of belonging. These events contributed to a positive work and social culture among community nursing teams, reinforcing their commitment to their roles and the organization.

### Theme 3: alignment with organizational values

The culture and values of an organization contributed towards attracting, but in particular, retaining community nurses. Participants agreed that aligning with organizational values provided a sense of purpose and engagement, reinforced the organization’s goals and positively influenced decision-making.

#### Responsive organization

Permanent contract community nurses reported their commitment to their organizations due to proactive strategies to recruit and retain staff such as robust preceptorship programmes, clinical supervision, international recruitment initiatives, and offering conditional employment to student nurses before graduation.

*‘… attending the University before they qualify to explain to them what community nursing is, what is available, what support they have’.* (Morgan, clinical practice facilitator)

#### Organizational reputation

Participants’ decisions to join their organizations were influenced by public perception, the independent regulatory body ratings, media coverage and opinions of other community nurses through word of mouth. This sentiment was shared by most permanent contract participants. Some managers believed a good independent regulatory body rating, or national award recognition would attract nurses to join their organization.

*‘If the service is well led that also attracts staff to stay where they are … staff look around to see which Trust is good, bad in the CQC rating’.* (Jack, service manager)

Recommendations for joining an organization were based on family-oriented roles, professional development opportunities, and workplace flexibility.

*‘I knew someone working for them. She is the one who made me join actually and she spoke good things about the organisation because I wanted to go to school’.* (Chris, district nurse)

#### Being loyal to the National Health Service

Participants from all groups expressed positive views towards the National Health Service and attributing their continued employment to it. Some felt obligated due to the institution having supported their career growth, while others expressed commitment to the National Health Service constitution.

*‘… its NHS really… throughout my career from starting off in 1994, as a band 2 and progressing to a band 6 where I am now … they’ve invested in me, so, you know, I’m loyal to them…’* (Pat, district nurse).

### Theme 4: prior development and transitional experience

Community nurses reported developed a sense of identity and purpose during the experiential learning phase of being a community nurse. At this stage, they learned the role’s demands, developed a professional identity and identified areas for growth. More significantly, they also discovered the most rewarding aspects of the role.

*‘The reason I joined the team that I did was because I had a student placement there and I really enjoyed going there …’* (Mel, community staff nurse).

Placement experiences helped students understand that community nursing involves more than just single administered tasks, it gave a sense of nursing skills needed to meet the complex needs of patients at home. Some participants initially believed that community nursing was slow-paced and only suitable for nurses nearing retirement; they talked about deskilling. Experience debunked these misconceptions, leading them to pursue full-time community nursing roles as their first jobs post-registration.

### Theme 5: job dissatisfaction

Dissatisfaction with the job has led some nurses to consider leaving their positions. Six distinct sub-themes related to job dissatisfaction in community nursing were identified from the data.

#### Staffing shortage

All participants acknowledged that these shortages were influenced by challenges in recruitment and retention, as well as a lack of skill mix and succession planning. Shortages led to less experienced staff being appointed to more senior roles for which they were not able or qualified. This was perceived as unsafe and raised concerns about professional registration. Some temporary participants said they would decline shifts from certain teams due to perceived unsafe practices.

*‘It was very stressful because one day you go to work instead of six nurses, you realise you have only three nurses with about 100 visits to do on that day’.* (Paris, district nurse)

#### Lack of career progression

Lack of career progression was reported by several participants with permanent contracts, both community nurses and service managers, as a reason for considering leaving community nursing. This was due to the absence of opportunities; once a person had developed there was not always a gap in need in more senior roles. The banding system was perceived by participants in the coastal and rural organizations as unfair. In these organizations, a junior community nurse had to complete district nurse training to progress to a higher band. Compared with community nurses in the urban organization who could apply for a higher band without this qualification. Service managers from rural and coastal organizations reported that they were losing community nurses to primary care and commissioning bodies.

*‘We have lost a lot of community nurses to go to [primary care] … we [have] limited amount of resource where [they] have got a bit more’.* (Stella, service manager)

#### Feeling exploited

There was a consensus among participants that there was an unwritten cultural and professional expectation of goodwill, willingness and acceptance among community nurses, which could lead to exploitation, such as being underpaid and working unpaid overtime. This further contributed to job dissatisfaction.

*‘There is a very much a culture of expectation that people will go above and beyond … I really believe that is why a lot of people end up leaving’.* (Faye, district nurse)

Some service managers acknowledged that community nurses often worked beyond their contracted hours.

*‘Maybe the organisation takes a bit of an advantage of our staff. But at the same time, they do not put anyone in the position to actually do it. Staff just feel that they need to do it because they are not going to leave their patients like that’.* (Stevie, service manager)

There were reported disparities in overtime remuneration across the three organizations. While service managers claimed there were clear remuneration processes, community nurses with permanent contracts reported that overtime pay was discretionary and not guaranteed. Temporary contract holders from the urban organization sometimes stopped pursuing overtime payments due to the complicated process and difficulties in getting their timesheets signed.

#### Steppingstone

Participants with permanent contracts across organizations were using community nursing as a stepping stone to other roles. These nurses were described as opportunistic, seeking employment to access developmental opportunities, even if their ultimate goal was not to remain in community nursing. Newly qualified nurses sometimes took community nursing roles while awaiting opportunities in other nursing fields.

*‘Two guys left straight away only because they used it as a stepping stone for something else they left within weeks. They wanted jobs in A&E [the emergency department] and could not get it at the time’.* (Ash, clinical practice facilitator)

#### Disillusionment

Many senior participants believed that some nurses leave community roles due to disillusionment stemming from unrealistic expectations of the job. For some this included having less personal autonomy than expected whereas others experienced anxiety on realizing the decision-making responsibilities expected.

*‘They think it’s an easier job … go into people’s homes … cups of tea … chat … they have their eyes opened’.* (Morgan, clinical practice facilitator)

#### Lack of identity

Temporary contract nurses from the urban organization felt anonymous. They were often referred to as ‘agency nurse’ rather than by their names, despite weeks of service. These nurses said they were perceived as less caring and money-driven, with assumptions that they earned more than permanent staff. These attitudes from permanent staff reportedly deterred them from considering permanent positions in community nursing.

*‘I do not see myself as an agency nurse…I have a name. [They say] agency nurse did this, agency nurse did that … badmouthing only the agency nurse’.* (Blake, community staff nurse)

### Theme 6: shift in traditional practices

Changes in the need and manner of administration, care delivery, fund availability and some technologies caused some participants to question their reasons for joining community nursing and consider leaving.

#### Increased administration

Many participants from all organizations reported that increased administration led them to consider leaving their jobs. Many joined community nursing for the freedom to provide individual care in homes or clinics. However, increased administrative tasks, including report writing and incident investigations led to reduced patient contact.

*‘It used to be when I took on this role, I would have one management day a week. And the rest of the days, I would be out seeing patients … I have lost that connection … the only time now that I deal with patients is when it is something negative …a complaint or a concern’.* (Fiona, district nurse)

Increased administration resulted in longer working days and lost family time. Managers recognized the increased administrative tasks and extra hours put in by community nurses and tried to promote work-life balance. This attempt was challenged by the need to complete tasks and meet targets.

#### Task focused

Many participants perceived nursing has become more task-focused, deviating from a patient-centred, holistic approach. A shift, influenced by heavier workloads and patient complexity, they believed had negatively impacted care delivery.

*‘it’s become so task orientated. We tend to just go in and do the task and not see the bigger picture’.* (Dom, district nurse)

Many mid-career staff, particularly in rural and coastal areas were dissatisfied their role had shifted to triaging patients rather than providing direct care. They felt they lost the personal connection that attracted them to community nursing. Two mid-career staff planned to leave for non-clinical roles due to disagreement with this direction and what they perceived as modern values of community nursing.

*‘You are literally chasing visits … not looking … your chart is in place … I left district nursing for specialist nursing’* (Daryl, community specialist nurse).

#### Financial constraints

Most participants noted financial limitations within community nursing groups. These constraints were perceived to being commissioners demanding more return on their investment through targets and other activity-related goals, often without budget increases and sometimes with reduced funding. Consequently, nursing providers were continually seeking cost-saving measures, often this was perceived to be at the expense of service quality and patient outcomes.

*‘They are more worried about, you know figures and you know, KPIs [key performance indicators] … rather than, you know what is actually happening’.* (Sue, service manager)

Managers from all three organizations acknowledged that financial constraints sometimes led to not replacing departing community nurses as a cost-saving measure. They recognized that this approach increased the workload and pressure due to staff shortages.

#### Electronic documentation

Organizations were transitioning from paper to electronic records for easier access to patient data during home visits. Although participants appreciated the need for the shift, many were unfamiliar with the relevant systems and in some cases, they needed to fill out different templates on various platforms. Some nurses nearing retirement opted for early retirement rather than adopt or learn the technology.

*‘It doesn’t work for us, it has made our jobs harder’* (Naomi, community staff nurse).

Most nurses in all organizations reported unreliable connectivity, especially in patients’ homes with no or weak internet access. Nurses were required to complete electronic documentation within 24 hours of patient contact which caused anxiety. As a result, they often completed their visits and then input patient data at home in the evening. There was a consensus that the shift to electronic documentation had led to increased workload.

*‘Since they introduced a new computer system, staff not just myself but other staff have been working two or three hours overtime to try and document everything’.* (Dom, district nurse)

### Theme 7: lack of compassionate leadership

Organizational culture and leadership practices significantly influenced community nurses’ decisions to leave or consider leaving their jobs. They perceived lack of empathy and disconnect from people in leadership positions.

#### Lack of support

Community nurses, regardless of contract type, reported a lack of managerial or organizational support. Some shared experiences of managers agreeing to personal or work-related arrangements (e.g. flexible working) but failing to do so. Others felt their managers considered them inefficient when they failed to complete their work in contracted hours.

*‘She thinks sometimes that you could’ve done that during your day, so I just don’t claim it’.* (Bobby, community staff nurse)

Others reported unprofessional behaviour from service managers such as public reprimands. This had led to some leaving for other teams or organizations. Some went as far as to say they were bullied.

*‘There was a time when I did consider leaving here due to bullying, which was raised and it took time obviously, procedures and policies have to be followed. It was the people around me that actually made me feel that I can actually do my job and it wasn’t me’.* (Paris, district nurse)

Community nurses from all three organizations reported a lack of support from their service managers which they attributed to the managers’ limited knowledge and experience in adult community nursing. These managers were reported to agree to unrealistic or unachievable expectations with commissioners or patient families.

#### Poor communication

Participants from all organizations, regardless of their contract type, reported poor communication within their work environments. This was primarily seen as a lack of information about new systems and a lack of consultation and involvement in the implementation of systems that impacted their daily work activities.

*‘You just see something, and you are like when did this start? Then you start calling around trying to find out who started this thing’.* (Al, district nurse)

Poor communication was exacerbated by cliques of staff or what was seen as ‘organisational politics’ where certain opinions were prioritized over others. This led to team divisions resulting in temporary community nurses disinclined to accept permanent positions, and newly qualified nurses considered leaving the team.

*‘There is a lot of politics to be honest. At times you cannot really express yourself. You have to be very careful [what you say]’.* (Ricki, community staff nurse)

### Theme 8: family commitments

Although the perception of flexibility in the community nurse role attracted some, this factor did not always result in retention. Working hours led some to consider leaving the role.

*‘I’ve got to pick Joey up at 4 o’clock … my mom’s just phoned, she’s had a fall and my husband’s just made redundant. The stuff that people deal with on a day to day basis, they’re parts of the skills of a manager is to try and help people to deal with that and to move on with it and to try and… and to live with those crises…’* (Sue, service manager).

Family commitments, viewed as caring responsibilities towards family members, children, and parents, significantly influenced community nurses’ work decisions with some considering reducing their work hours. Some nurses in rural areas relocated to be closer to aging parents, leading to a shift from one community nursing organization to another.

## Discussion

The findings from this study highlight the factors influencing nurses’ decisions to pursue and remain in community nursing roles, aligning well with Herzberg’s Two-Factor Theory of Motivation and Hygiene factors (Herzberg, 1967). The participants expressed strong connections to both motivators (intrinsic job satisfaction) and hygiene factors (extrinsic job conditions), which impacted their engagement, retention, and career progression within community nursing. Various motivational theories address employee motivation (Pardee, 1990), but their perspectives may not fully align with data on community nurses’ recruitment and retention. Maslow’s Hierarchy of Needs, for example, suggests employees have tiered needs driving motivation, culminating in self-actualization (Velmurugan and Sankar, 2017; More and Padmanabhan, 2017). However, this sequential model does not reflect findings from community nurses, who reported satisfying multiple needs simultaneously, for example, securing financial stability through employment while also achieving self-actualization through caregiving (Udechukwu, 2009). Additionally, Maslow’s theory primarily addresses recruitment rather than retention.

The theme of ‘The Perfect Job’ demonstrated that community nursing was perceived as an ideal career, particularly due to its emphasis on workplace flexibility and family orientation. Nurses valued the ability to balance personal and professional responsibilities, with workplace flexibility emerging as a significant hygiene factor that enhanced job satisfaction. According to Herzberg, job security and work-life balance play crucial roles in preventing dissatisfaction (Herzberg, 1967; Gardner, 1977; Hunt et al., 2012) which was reflected in participants’ appreciation of permanent contracts and service policies promoting adaptable working hours. This is consistent with broader nursing literature, which identifies flexibility as a key driver of nurse retention, particularly among those with caregiving responsibilities (Chenoweth et al., 2014; NHS Employers, 2022).

Participants also reported job autonomy as a defining feature of community nursing, falling under Herzberg’s category of motivators. Nurses emphasized their ability to make independent clinical decisions, manage complex patients, and provide person-centred care, which brought personal fulfilment and professional growth. Studies in nursing job satisfaction reinforce the importance of autonomy, suggesting that higher levels of clinical independence can improve work engagement and patient outcomes (Gottlieb et al., 2021; Pursio et al., 2024). However, some participants feared that reductions in autonomy could lead to work dissatisfaction and attrition, highlighting the delicate balance between empowerment and structural constraints within community nursing.

Another key motivator was the sense of belonging and alignment with organizational values, with nurses frequently referencing the supportive culture of community nursing. Feeling valued by colleagues, respected by patients, and aligned with the mission of the organization contributed to their job satisfaction and commitment. These findings align with Herzberg’s argument that a positive organizational culture and recognition enhance motivation (Ewen et al., 1966; Skripak et al., 2020). Furthermore, research suggests that nurses who experience a strong team culture and organizational alignment report greater job engagement and lower turnover rates (Wood et al., 2023; Yi et al., 2024).

Job security was another factor influencing career retention, with nurses expressing confidence in state-funded healthcare organizations providing financial stability. The perception that community nursing is appropriate for older staff also emerged, with experienced nurses appreciating the reduced physical demands compared to hospital work. These elements function as hygiene factors, ensuring that dissatisfaction is minimized, reinforcing existing findings that nurses value predictability and professional longevity when choosing to remain in their roles (Capper et al., 2020; Mlambo et al., 2021).

Interestingly, prior student placement experiences significantly shaped participants’ perceptions of community nursing. Exposure to real-world patient care in home settings allowed nurses to appreciate the complexity and significance of their role beyond traditional hospital environments. These findings echo literature on nursing career transitions, which suggests that experiential learning fosters professional identity development and long-term career commitment (Kerr and Macaskill, 2020; Aldosari et al., 2021).

Staffing shortages emerged as one of the most pressing concerns, with participants citing high workloads, insufficient skill mix, and gaps in succession planning. Nurses described overwhelming schedules, which impacted their ability to provide safe and effective care. Herzberg’s theory suggests that working conditions and organizational policies are crucial hygiene factors that influence retention (Hackman, 1980; Skripak et al., 2020), when nurses perceive the work environment as unsafe or poorly structured, dissatisfaction increases (Senek et al., 2020; Dall’Ora et al., 2015). This is consistent with wider nursing literature, which identifies nursing shortages as a key contributor to burnout and turnover (Ohue et al., 2021; International Council of Nurses, 2022).

Another major factor was lack of career progression, particularly among permanent contract nurses and service managers. Participants expressed frustration with limited advancement opportunities and inconsistencies in the banding system, particularly in rural and coastal areas, where additional qualifications were required for promotion. Herzberg emphasizes that opportunities for growth and recognition are essential motivators, and when absent, dissatisfaction leads to attrition. Research confirms that nurses are more likely to leave roles with unclear progression pathways (Royal College of Nursing, 2025; Stoye and Warner, 2024).

The theme of feeling exploited highlighted issues of unpaid overtime and an informal expectation to go beyond contracted hours. Some participants believed this was due to cultural norms within nursing, while others felt their organizations took advantage of their goodwill. Herzberg categorizes salary and fair policies as hygiene factors. It is likely when nurses perceive disparities in overtime pay or lack of recognition, dissatisfaction deepens (NHS Employers, 2024). Studies on nurse retention reinforce that fair compensation and well-defined workload expectations are essential for job satisfaction and workplace morale (Poku et al., 2025).

Additionally, community nursing was sometimes viewed as a stepping stone, with participants reporting that some nurses took temporary roles while awaiting preferred positions elsewhere. This opportunistic career approach suggests that community nursing may struggle with long-term retention, particularly for newly qualified nurses. Literature on nurse career development suggests that retention improves when organizations provide structured mentoring and professional identity development initiatives (Hoover et al., 2020).

Disillusionment emerged as another concern, with some nurses experiencing misconceptions about the role before joining. Some believed community nursing would offer greater autonomy than reality, while others struggled with the complex decision-making responsibilities required in patient care. This aligns with Herzberg’s insight that work perception influences job satisfaction, and when expectations differ significantly from reality, role dissatisfaction increases (Mitsakis and Galanakis, 2022).

Lastly, temporary nurses reported feelings of anonymity and lack of identity, particularly when referred to as ‘agency nurses’ rather than their names. These nurses described a negative culture where permanent staff viewed them as transactional workers rather than valued team members. Herzberg categorizes organizational belonging and interpersonal relationships as motivators (Skripak et al., 2020), when nurses feel disconnected or undervalued, engagement declines (Poku et al., 2025). Research suggests that inclusive workplace cultures improve nurse retention by fostering strong peer relationships and professional validation (Adams et al., 2021).

Leadership and organizational culture play a crucial role in nurse retention, with findings indicating that lack of compassionate leadership and poor communication contribute to dissatisfaction. Additionally, while workplace flexibility is often seen as a benefit, family commitments sometimes lead nurses to reconsider their roles. These factors align with Herzberg’s hygiene factors, which, when inadequate (Herzberg, 1967; Hunt et al., 2012), contribute to workplace dissatisfaction and attrition.

Lack of managerial support was a recurring concern, with nurses reporting unfulfilled promises regarding flexible working arrangements and negative managerial attitudes towards workload management. Some participants described bullying and public reprimands, which significantly impacted their job satisfaction and mental well-being. Herzberg’s theory categorizes supervision and interpersonal relationships as hygiene factors (Skripak et al., 2020), when nurses perceive unsupportive leadership, dissatisfaction increases (Ofei et al., 2023). Research suggests that compassionate leadership improves nurse engagement and retention, reinforcing the need for empathetic managerial practices (West et al., 2022).

Poor communication further exacerbated dissatisfaction, with nurses citing lack of transparency in policy changes and exclusion from decision-making. Participants described organizational politics and cliques, which led to team divisions and reluctance among temporary nurses to accept permanent roles. Herzberg’s theory highlights effective communication and fair policies as essential hygiene factors, when absent, workplace morale declines (Nickerson, 2025). Studies confirm that open communication and inclusive leadership foster team cohesion and long-term retention (Carter, 2024; Ritu, 2024).

Family commitments also influenced career decisions, with some nurses considering reducing work hours or relocating to accommodate caregiving responsibilities. While workplace flexibility was initially seen as a motivator, it did not always result in long-term retention. Herzberg’s theory suggests that work-life balance and job security are hygiene factors (Skripak et al., 2020), when nurses struggle to balance personal responsibilities with professional demands, dissatisfaction increases (Senek et al., 2020; Smith, 2022). Literature on nurse workforce trends highlights that family commitments often drive career transitions, particularly among nurses in rural settings (WHO, 2021).

Overall, these findings affirm that intrinsic motivators and extrinsic hygiene factors interact to shape nurses’ perceptions of community nursing as a desirable career choice. Understanding these dynamics is critical for recruitment and retention strategies, ensuring that community nursing continues to be an attractive career path for both new and experienced professionals. Future research may further explore how organizational policies can better sustain motivation and satisfaction, particularly in the context of evolving healthcare demands.

### Implications for policy and practice

While no universal solution exists for the complex factors influencing community nurse recruitment and retention, organizations can take logical steps based on our findings. These include promoting workplace flexibility, training, and career progression opportunities. Recruitment materials should clearly communicate organizational values and achievements in patient care. Notably, nurses who had positive experiences during student placements in the community were more likely to apply for roles post-qualification. With higher education institutions increasing student nurse intake and facing placement challenges, this presents a valuable opportunity (Williamson et al., 2023).

### Strength and limitations

This qualitative study encompassed diverse workplaces across three distinct geographical areas, which enhances the breadth of the findings. However, the transferability of these results to different healthcare systems and care environments is limited. The study’s focus on community nursing within the English context may not fully capture the nuances and challenges faced by community nurses in other countries with different healthcare infrastructures and policies. Additionally, the reliance on self-reported data from participants introduces the potential for bias, as responses may be influenced by personal experiences and perceptions, especially with the study lacking diversity in ethnicity of participants. Future research should consider incorporating quantitative methods and expanding the scope to include a broader range of healthcare settings to validate and extend the findings.

### Alignment with existing literature

The findings of this study align with previous research highlighting workplace flexibility, autonomy, and organizational culture as key factors influencing nurse retention. Studies have consistently shown that autonomy in clinical decision-making enhances job satisfaction and professional engagement (Zychová et al., 2024; Şahan & Özdemir, 2023). Similarly, research on community nursing models, such as the Dutch Buurtzorg approach, emphasizes the importance of self-managed teams and patient-centred care, reinforcing the study’s findings on person-centred care and professional autonomy (Lalani et al., 2019).

Additionally, the study’s emphasis on job security and career progression aligns with broader nursing literature, which identifies predictability and professional development as critical for long-term retention (Wilson & Lucy, 2020). The European Federation of Nursing Associations has also highlighted workforce shortages and retention challenges, urging policy interventions to sustain nursing careers (De Raeve, 2022).

### Divergence from previous research

While previous studies have focused on reasons nurses leave, this study uniquely explores why nurses choose to stay, offering a fresh perspective on retention strategies. Traditional retention models often emphasize financial incentives and workload management (Chamanga et al., 2020), whereas this study highlights intrinsic motivators such as sense of belonging, alignment with organizational values, and professional identity.

Furthermore, the study challenges the assumption that community nursing is primarily attractive to older nurses. While previous research suggests that community nursing is a transition for aging professionals, this study finds that newly qualified nurses also view it as a viable career path, particularly when exposed to positive placement experiences.

### Theoretical contribution

By integrating Herzberg’s Two-Factor Theory, this study provides a structured framework for understanding nurse motivation and dissatisfaction. It expands on Herzberg’s hygiene factors by demonstrating that workplace flexibility, leadership support, and professional identity are equally critical in community nursing retention.

Additionally, the study contributes to evidence-based nursing practice by reinforcing the need for organizational policies that prioritize nurse well-being. Research on evidence synthesis in community nursing suggests that policy gaps and leadership deficiencies contribute to attrition, reinforcing the study’s findings on poor communication and lack of compassionate leadership (Kennedy and Ramukumba, 2020).

### Recommendations for further research

Our study is among the few qualitative investigations into community nurse recruitment and retention, highlighting the need for further research. Many challenges identified by participants could be addressed with straightforward solutions. We recommend co-design processes to collaboratively develop effective strategies.

## Conclusion

This study highlights the multifaceted challenges surrounding community nurse recruitment and retention, emphasizing the need for targeted strategies that go beyond traditional hospital-focused approaches. While salary improvements remain crucial, broader systemic changes including workplace flexibility, compassionate leadership, and career development opportunities are essential to fostering a sustainable workforce. By addressing these factors through co-designed solutions and evidence-based policy adjustments, healthcare organizations can enhance job satisfaction, reduce attrition, and ultimately strengthen the future of community nursing. With further qualitative research, these insights can inform lasting reforms that support the evolving demands of community nursing.

## Data Availability

Research participants for this study did not consent for their data to be made available to other parties other than the research team.
